# Automatic weighing system vs. manual weighing precision comparison in PM-loaded filter measurements under different humidity conditions

**DOI:** 10.1007/s10661-023-11939-7

**Published:** 2023-10-31

**Authors:** Dmytro Chyzhykov, Kamila Widziewicz-Rzońca, Monika Błaszczak, Patrycja Rogula-Kopiec, Krzysztof Słaby

**Affiliations:** Institute of Environmental Engineering, Polish Academy of Sciences, 34 M. Skłodowskiej-Curie St, 41-819 Zabrze, Poland

**Keywords:** Particulate matter, PM mass measurement, Gravimetric analyses, Robotic weighing, Manual balances

## Abstract

Currently, there is a lack of research directly comparing the precision of automatic weighing systems and manual weighing in the context of particulate matter (PM) filter equilibration and measurements under different humidity conditions. During experimental measurements, three different types of PM-loaded filters were weighed using manual and automatic balances. During manual weighing, every filter was weighed twice in three different relative humidity conditions. The same procedure was done using an automated weighing system. In most cases, it was found that under relative humidities in the range of 30–55% RH, the manual and automated methods can be treated as referential. Regarding device stability, very slight but overall better precision was found for 30% RH, suggesting that 40 CFR Part 50, Appendix L requirements regarding conditioning humidity (30–40% RH) seem more suitable than those presented in the PN-EN 12341:2014 standard (45–50% RH). Understanding the effects of the influence of the RH% on PM mass measurements is a matter of great importance, because water vapor condensed on a filter can affect the particulate matter concentrations. This is especially important in areas where regulatory limits are exceeded. Calculation of uncertainty in the PM mass measurements is therefore crucial for determining the actual sample mass and improving air monitoring practices. In a nutshell, the experimental results obtained clearly describe how changing RH% conditions affect the PM weighing precision during manual and automated measurements.

**Supplementary Information:**

The online version contains supplementary material available at 10.1007/s10661-023-11939-7.

## Introduction

In our investigation, we sought to examine the precision of manual and automated mass measurements in the context of weighing particulate matter (PM). By objectively assessing the precision of manual and automated mass measurements, our research provides valuable insights into the potential role of robotic weighing systems in routine air monitoring. The impact of particulate matter (PM) on human health and the environment is a critical issue that necessitates thorough study and effective mitigation strategies. Studies show that exposure to PM in ambient air has been linked to a number of different health outcomes, including lung inflammatory reactions (Jia et al., 2021; He et al., 2017), respiratory symptoms (Hu et al., 2022; Xing et al., 2016), adverse effects on the cardiovascular system (Czernych et al., 2023; Qu et al., 2018; Yang et al., 2021), an increase in chronic obstructive pulmonary disease (Duan et al., 2023; Park et al., 2021), reduction in lung function in adults (Bo et al., 2021), and a reduction in life expectancy (Apte et al., 2018) owing mainly to cardiopulmonary mortality and probably to lung cancer (World Health Organization Europe, 2006). As particulate matter concentration levels are an important subject of observation and attention, this fact underscores the continued demand for a thorough and precise study of this issue.

Accurate measurement of particulate matter involves employing robust methodologies and instrumentation. This is strictly connected with lowering uncertainties in PM mass measurements (Lacey & Faulkner, 2015), especially in the case of gravimetric analyses of fine and ultrafine particles. Buonanno et al. (2011) calculate the average relative uncertainties related to the gravimetric measurement of each fraction of PM as follows: 8% for PM10, 13% for PM2.5, and 14% for PM1. Notably, these results reveal an increasing relative uncertainty as the measured PM fraction decreases.

In this research, we are trying to concentrate on the measurements of filters loaded with particulate matter under sets of different relative humidity conditions with the use of a traditional manual balance and a robotic weighing system. The currently existing standards provide general specifications regarding the conditioning of filters: the EN 12341:2014 standard (EN 12341, 2014) specifies conditioning at 19–21°C ± 2°C and 45–50 ± 5% RH and the 40 CFR Part 50, Appendix L standard (40 CFR Appendix L to Part 50 n.d) provides such specifications regarding the conditioning of PM_2.5_ sample filters at 20–23°C ± 2°C and 30–40 ± 5% relative humidity, However, the seemingly simple gravimetric analysis procedure is subject to influence from multiple factors including laboratory effects (e.g., temperature and relative humidity [RH] fluctuations in the weighing environment, dust contamination, static charge effects) and non-laboratory activities (e.g., volatilization of sampled PM, decrease in filter mass due to accidental loss of PM from the filter surface during transportation) impacting the measured mass of the particles deposited on the filter. Eliminating and/or minimizing these interferences during pre-sampling and post-sampling weighing is critical for a precise calculation of the PM net mass loading and subsequent mass concentration calculation (Presler-Jur et al., 2016).

Moreover, questions have been raised about the way that temperature and relative humidity cause mass deviations and the best technique to minimize those effects (Widziewicz-Rzonca et al., 2020; Widziewicz-Rzońca et al., 2022). Studies by Barba-Lobo et al. (2022) suggest the use of a new and simple methodology to calculate uncertainty and accurately determine the mass of the particulate matter deposited onto sampled PM filters, by using a so-called control filter, which is always exposed to the environmental conditions present in the laboratory. Another study (Presler-Jur et al., 2016) compared manual and robotic weighing systems and proved that the robotic weighing system had a high degree of precision and accuracy. This was evident from the lack of any change in the filter weight for repetitive weighing of a single filter over a 3-day period (2 μg standard deviation) and no change in standard weights (metal reference). While comparing robotic and manual weighing, the robotic weighing system was found to be minimally impacted by static. Laboratory blank results also indicate that there is no additional risk of debris contamination for robotically weighed filters relative to manually weighed filters (Presler-Jur et al., 2016). While there are a number of studies related to the influence of effects of static on PM filters (Chase et al., 2005; Ji et al., 2023; Swanson & Kittelson, 2008), none provide an understanding of which technique minimizes the influence of the temperature and relative humidity on PM mass measurements. In order to gain some knowledge in this area, we investigate the influence of relative humidity on PM measurements by performing manual and robotic weighing for PM mass measurements under three different RH conditions. The comparison of the two measuring techniques, manual and robotic, under different RH will bring an understanding of which technique best minimizes those effects during measurements.

The main purpose of the study was to determine how changing conditions of humidity influence the precision of weighing during robotic and manual measurements of PM-loaded filters. A comprehensive understanding of the influence of humidity on particulate matter in both manual and robotic measurement procedures is a significant concern, warranting further investigation. Also PM fractionation is a factor influencing the hygroscopicity of filters (Tian et al., 2016; Wang et al., 2020), which should be taken into account regarding the repeatability of mass measurements.

## Materials and methods

### Filters conditioning, weighing, and weighing environment

**Fig. 1:**
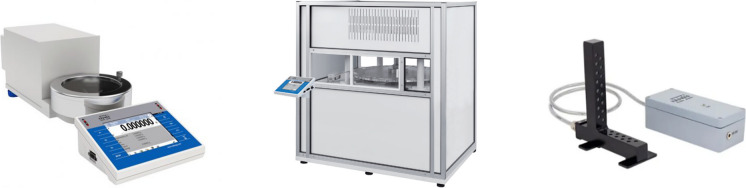
Manual balance MYA 5.4Y.F (Radwag, Microweight MYA 4Y.F, http://www.radwag.com), automatic weighing system UMA 2.5Y.FC (Radwag, Automatic Weighing System UMA 2.5Y.FC. https://radwag.com/pl/automatyczny-system-wagowy-uma-2-4y-fc,w1,8J8,401-190), and DJ-05 antistatic ionizer (Radwag.com)

In this research, mass measurements were performed using the manual weighing device MYA 5.4Y.F as well as the UMA 2.5Y.FC robotic weighing system—RWS (Fig. 1) produced and distributed by Radwag Balances and Scales (http://radwag.com, Radom, Poland). Also, a DJ-05 antistatic ionizer (Radwag) was used to minimize the effect of static on the filters during measurements. The manual weighing device MYA 5.4Y.F, according to its metrological specifications, has a maximum capacity of 5.1 g and a readability of 1 μg. The robotic weighing system (RWS) UMA 2.5Y.FC, according to its metrological parameters, has a maximum capacity of 2.1 g and readability of 1 μg (http://radwag.com). Prior to manual measurements, the filters were equilibrated in a weighing room where humidity conditions were maintained by the automatic humidifier/dehumidifier HB CCS0401S (HB Polska Sp. z o.o., https://hbpolska.pl/) characterized by an operating range of 30–80% RH. Registration of temperature and humidity conditions during manual measurements was done by a Q-MSystem Module (POL-LAB, Pol-Lab.eu) with the following accuracy of temperature and humidity measurements: ± 0.5°C and ± 3% RH, with the resolution of temperature and humidity, 0.1°C and 0.1% RH, respectively.

The RWS has a built-in environmental chamber which guarantees constant temperature and relative humidity conditions. This feature ensures that conditions for filter equilibration will not fluctuate and will be appropriate in accordance with the EN 12341:2014 standard. During both conditioning and weighing, the robotic system maintained constant ambient conditions in the chamber with stability of ± 1°C operating temperature and RH stability ± 2.5%. During robotic measurements, the temperature and humidity conditions inside the chamber were registered automatically with the use of in-built sensors with a temperature measurement resolution of 0.001°C and humidity measurement resolution of 0.001% (UMA 2.5Y.FC, Radwag). Before the measurements, each filter was examined for any damage (e.g., wetting of the edge of the filter, holes, or any other visible damage, or loss of its flat form) that might cause unstable mass measurements.

Following the EN12341:2014 standard, the filters were subjected to dual weighing sessions with a minimum 12-h interval before exposure. This procedure ensured the stability of the filter weight. If the weight discrepancies for non-loaded filters exceeded 40 μg for LVS, the respective filter was deemed unsuitable and excluded from analysis. Post-exposure, the filters underwent another two rounds of weighing. After being placed in the weighing laboratory or weighing device (in the case of robotic measurements) for 48 h, they were measured twice on a balance. Results deviating by more than 60 μg were disregarded. This led to a reduction in the number of observations compared to the initial 15 measurements, as vividly depicted by the distributions presented in the Appendix (supplementary materials). The total mass of each filter is an average from two weighings. Management of the weighing process, such as calibration during the robotic measurements, was performed by RMCS software as well as during manual measurements.

Trials were carried out for three types of filters: glass (G) and quartz fiber (Q) and PTFE (polytetrafluoroethylene) filters supported with a PTFE O-ring (Table 1).

**Table 1:** Characteristics of Whatman® filters used in mass measurements

No.	Filter type	Product no.	Pore size [μm]	Whatman grade	Thickness [μm]	Water retention	Filter effectiveness [%]
1.	Glass fiber	1820-047	1.6	GF/A	220	*Nd.	98
2.	Quartz fiber	1851-047	2.2	QMA	450	*Nd.	98
3.	PTFE O-ring**	7592-104	2.0	*Nd.	30–50	Mass addition <10 μg^1)^	99.7

*Nd. no data

**PTFE (polytetrafluoroethylene) with support ring for PM_2.5_

^1^After 24h of exposure under 40% RH in relation to the mass addition after 24h of exposure under 35%

Measurements were carried out for glass (G) and quartz (Q) filters loaded with two different fractions of particulate matter: PM_1_ (particles with aerodynamic diameter < 1 μm) and PM_2.5_ (particles with aerodynamic diameter <2.5 μm), but also for PTFE loaded with PM_2.5_, characterized by a pore diameter of 2.0 μm for PM_2.5_ collection. Fifteen samples of each type were collected. The filters were conditioned for 24 h before the weighing process under conditions of 30%, 45%, and 55% RH and in the temperature range specified in the PN-EN 12341:2014 standard. Actual measurements of the filter mass were performed under the same relative humidity values of 30%, 45%, and 55% RH and the same temperature range that were kept automatically in the RWS and the room where the microbalance was located. Particles were collected using a low volume (2.3 m^3^/h) Micro PNS Type LVS16c (Umwelttechnik MCZ GmbH, Germany) equipped with two sampling heads (PM_1_ and PM_2.5_). Samples were taken at the Zabrze measurement site during the winter period 24.11.2022–06.02.2023. Simultaneously, meteorological parameters at the measurement site were collected as well as the temperature and humidity inside the sampler airstream.

The application of RMCS filters in the measurements enabled the compensation of air buoyancy. A correction factor was applied after entering the air density value and the known density of filters into the balance’s memory. Subsequent to inputting these values, the program automatically computed the correction factor for the weighed filters and displayed their corrected mass.

### Statistical analysis

For statistical analyses, Statistica 13 software (StatSoft, Kraków) was used. These included testing the normality of the PM mass distribution using the Shapiro–Wilk test (*p*<0.05) (Appendix 1), and preparation of descriptive statistics (Table 2). When the probability was greater than the significance level, the distribution was treated as log-normal (Appendix 1, Figs. A1–A9). After retrieving all the measurement results of each group of filters of one type, the standard deviation was calculated using the following formula:

1 $$\boldsymbol{s}=\sqrt{\frac{\sum_{\boldsymbol{i}=\textbf{1}}^{\boldsymbol{n}}{\left({\boldsymbol{x}}_{\boldsymbol{i}}-\overline{\boldsymbol{x}}\right)}^{\textbf{2}}}{\left(\boldsymbol{n}-\textbf{1}\right)}}$$

where:

**Table 2:** Descriptive statistics for 10 repeatable measurements (both manual and robotic) of standard mass piece under different RH% conditions

Variable	*N*	Mean [g]	Min [g]	Max [g]	Std. dev [g]	Coeff. var. [%]
RH=30%						
Mass manual	10	0.149814	0.149812	0.149816	0.000001	0.000821
Mass robotic	10	0.149815	0.149814	0.149816	0.000001	0.000451
RH=45%						
Mass manual	10	0.149813	0.149812	0.149816	0.000001	0.000858
Mass robotic	10	0.149816	0.149815	0.149817	0.000001	0.000444
RH=55%						
Mass manual	10	0.1498138	0.149811	0.149819	0.000002	0.001364
Mass robotic	10	0.1498174	0.149815	0.149819	0.000001	0.000844

**Fig. 2:**
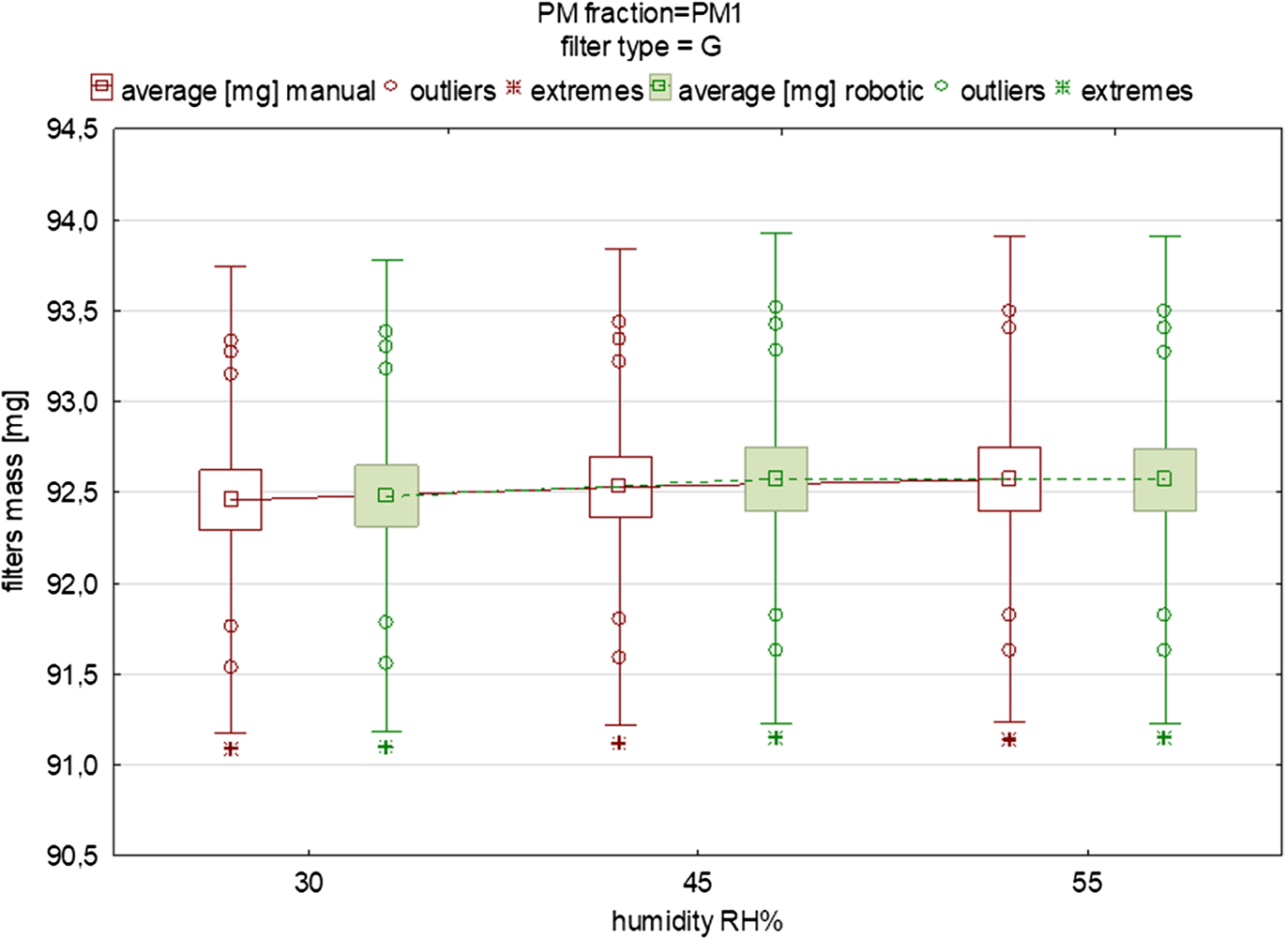
The influence of variable equilibration humidity on the mean mass and deviations of glass filters loaded with PM_1_ (each box was drawn based on measurements of 15 filters from one batch)

**Fig. 3:**
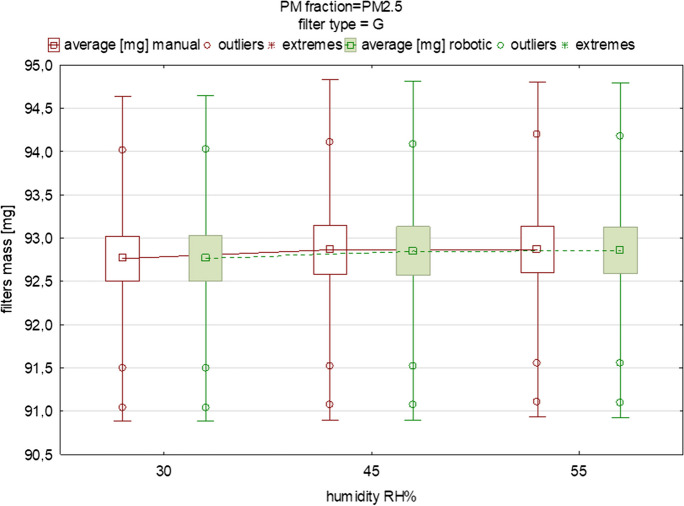
The influence of variable equilibration humidity on the mean mass and deviations of glass filters loaded with PM_2.5_ (each box was drawn based on measurements of 15 filters from one batch)

**Fig. 4:**
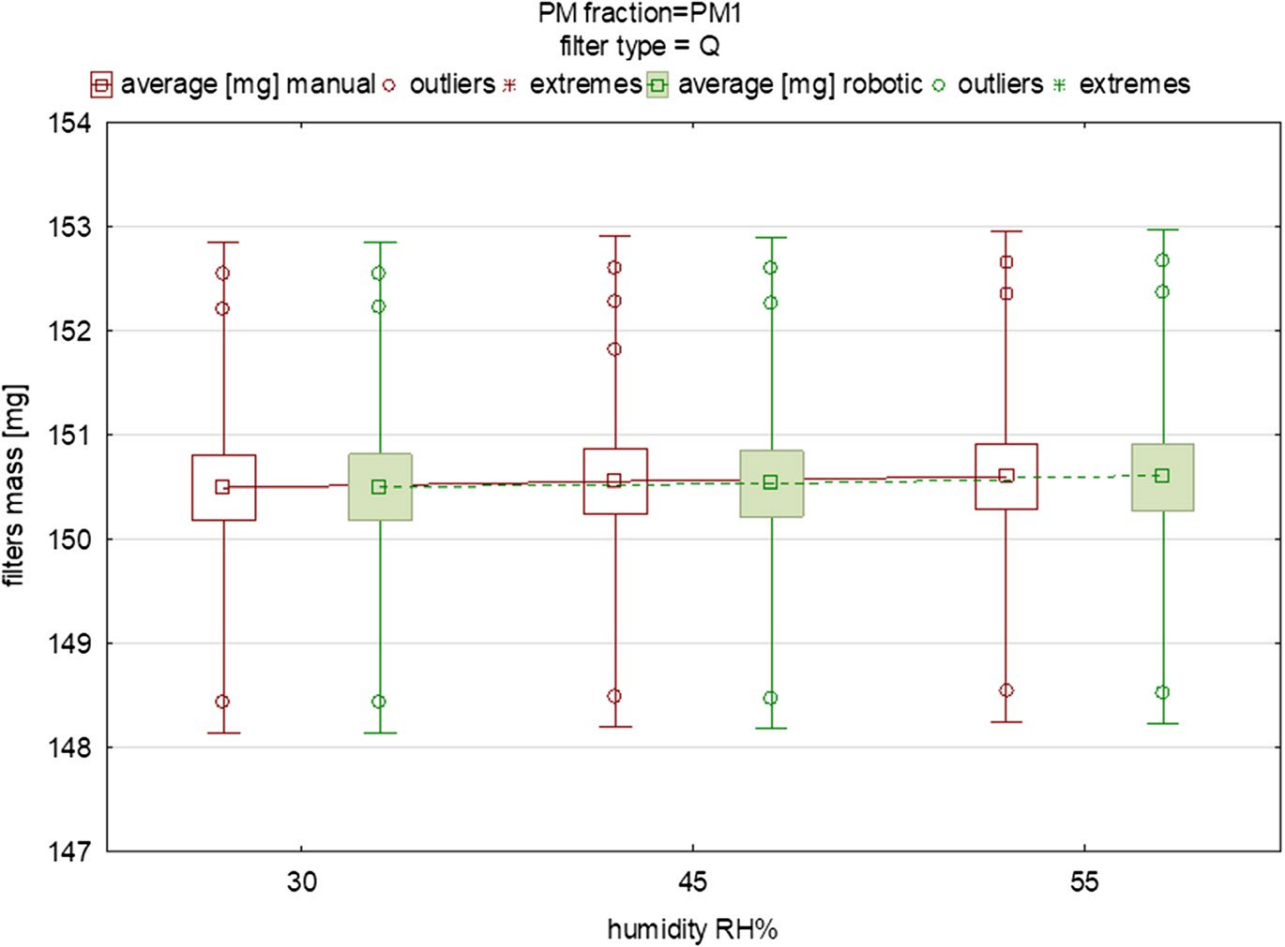
The influence of variable equilibration humidity on the mean mass and deviations of quartz filters loaded with PM_1_ (each box was drawn based on measurements of 15 filters from the one batch)

*n*—number of repetitions (measurements),

*x*_*i*_—result of the mass measurement, and

*x*—average value for *n* repeated mass measurements.

The effect of the type of mass measurement on the outcome variable—the filter mass—was tested using Student’s *t*-test for dependent groups (*p*<0.05) for each humidity tested (Tables 3, 4, 5, 6, 7). The procedure calculates the differences between the values of the two variables for each observation and tests whether the averages differ from “0.” Observations of each pair of data were made under equal conditions and the differences in means were normally distributed. This test is characterized by the degrees of freedom df = (*N*−1). Differences between the number of data in each of the humidity conditions resulted from some cases where the differences between the first and second mass measurements after exposure were greater than 60 μg. In these cases, the data were not included in the final dataset. The deviations in filter mass resulting from relative humidity variations were visualized using box-plot graphs (including different types of filter media, PM fractionation, and the influence of humidity on mass deviations).

**Table 3:** Descriptive statistics for 10 repeatable measurements (both manual and robotic) for standard mass piece together with reference filters under different RH% conditions

Variable	*N*	Mean [g]	Min [g]	Max [g]	Std. dev [g]	Coeff. var. [%]
Glass filters RH=30%						
Mass manual	10	0.241086	0.241082	0.241091	0.000003	0.001128
Mass robotic	10	0.241063	0.241062	0.241064	0.000001	0.000339
Quartz filters RH=30%						
Mass manual	10	0.294439	0.294434	0.294447	0.000004	0.001328
Mass robotic	10	0.294405	0.294402	0.294415	0.000004	0.001344
PTFE O-ring RH=30%						
Mass manual	10	0.284682	0.284674	0.284685	0.000003	0.001162
Mass robotic	10	0.284687	0.284686	0.284688	0.000001	0.000222
Glass filters RH=45%						
Mass manual	10	0.241069	0.241064	0.241074	0.000004	0.001749
Mass robotic	10	0.241066	0.241065	0.241069	0.000001	0.000428
Quartz filters RH=45%						
Mass manual	10	0.294422	0.294417	0.294426	0.000003	0.001060
Mass robotic	10	0.294411	0.294409	0.294418	0.000003	0.000906
PTFE O-ring RH=45%						
Mass manual	10	0.284665	0.284662	0.284667	0.000002	0.000688
Mass robotic	10	0.284785	0.284778	0.284789	0.000003	0.001100
Glass filters RH=55%						
Mass manual	10	0.241083	0.241081	0.241084	0.000001	0.000353
Mass robotic	10	0.241066	0.241065	0.241069	0.000001	0.000428
Quartz filters RH=55%						
Mass manual	10	0.294462	0.294458	0.294465	0.000002	0.000785
Mass robotic	10	0.294411	0.294409	0.294418	0.000003	0.000906
PTFE O-ring RH=55%						
Mass manual	10	0.284682	0.284679	0.284684	0.000002	0.000549
Mass robotic	10	0.284785	0.284778	0.284789	0.000003	0.001100

**Table 4:** Results from Student’s *t*-test for dependent samples for testing significance of differences in average filter mass between manual and automatic measurement (glass filters, PM_1_)

Variable	Mean [mg]	Std. dev. [mg]	*N*	Mean diff. [mg]	Std. dev. diff. [mg]	*t*	df	*p*	Conf. int. −95%	Conf. int. +95%
PM fraction=PM_1_, humidity=30% glass filters										
Mass manual	92.46	0.64	15	−0.02	0.33	−0.28	14	0.4653	0.7818	−0.21
Mass robotic	92.48	0.65								
PM fraction=PM_1_, humidity=45% glass filters										
Mass manual	92.53	0.66	15	−0.05	0.33	−0.54	14	0.5950	−0.23	0.14
Mass robotic	92.58	0.67								
PM fraction=PM_1_, humidity=55% glass filters										
Mass manual	92.57	0.67	15	0.00	0.32	0.05	14	0.9638	−0.17	0.18
Mass robotic	92.57	0.67								

bolded values are significant with *p*<0.05

**Table 5:** Results from Student’s *t*-test for dependent samples for testing significance of differences in average filter mass between manual and automatic measurement (glass filters, PM_2.5_)

Variable	Mean [mg]	Std. dev. [mg]	*N*	Mean diff. [mg]	Std. dev. diff. [mg]	*t*	df	*p*	Conf. int. −95%	Conf. int. +95%
PM fraction=PM_2.5_, humidity=30% glass filters										
Mass manual	92.76	0.94	13	−0.01	0.01	−2.18	12	**0.0495**	−0.01	−0.00
Mass robotic	92.77	0.94								
PM fraction=PM_2.5_, humidity=45% glass filters										
Mass manual	92.87	0.98	12	0.01	0.01	7.09	11	**0.0000**	0.01	0.02
Mass robotic	92.85	0.98								
PM fraction=PM_2.5_, humidity=55% glass filters										
Mass manual	92.87	0.97	13	0.01	0.01	2.69	12	**0.0196**	0.00	0.02
Mass robotic	92.86	0.97								

bolded values are significant with *p*<0.05

**Table 6:** Results from Student’s *t*-test for dependent samples for testing significance of differences in average filter mass between manual and automatic measurement (quartz filters, PM_1_)

Variable	Mean [mg]	Std. dev. [mg]	*N*	Mean diff. [mg]	Std. dev. diff. [mg]	*t*	df	*p*	Conf. int. −95%	Conf. int. +95%
PM fraction=PM_1_, humidity=30% quartz filters										
Mass manual	150.49	1.18	14	−0.00	0.143	−0.11	13	0.9132	−0.08	0.07
Mass robotic	150.49	1.18								
PM fraction=PM_1_, humidity=45%quartz filters										
Mass manual	150.55	1.18	14	0.02	0.01	6.52	13	**0.0002**	0.01	0.03
Mass robotic	150.53	1.18								
PM fraction=PM_1_, humidity=55% quartz filters										
Mass manual	150.59	1.18	14	0.00	0.12	0.02	13	0.9835	−0.07	0.07
Mass robotic	150.59	1.18								

bolded values are significant with *p*<0.05

**Table 7:** Results from Student’s *t*-test for dependent samples for testing significance of differences in average filter mass between manual and automatic measurement (quartz filters, PM_2.5_)

Variable	Mean [mg]	Std. dev. [mg]	*N*	Mean diff. [mg]	Std. dev. diff. [mg]	*t*	df	*p*	Conf. int. −95%	Conf. int. +95%
PM fraction=PM_2.5_, humidity=30% quartz filters										
Mass manual	151.128	0.88	11	−0.11	0.36	−1.02	10	0.3295	−0.35	0.13
Mass robotic	151.239	0.78								
PM fraction=PM_2.5_, humidity=45% quartz filters										
Mass manual	151.31	0.79	11	0.03	0.02	6.02	10	**0.0001**	0.02	0.04
Mass robotic	151.28	0.79								
PM fraction=PM_2.5_, humidity=55% quartz filters										
Mass manual	151.35	0.79	11	−0.00	0.02	−0.52	10	0.6152	−0.01	0.01
Mass robotic	151.36	0.81								

bolded values are significant with *p*<0.05

### Reference filter and standard mass measurements

Furthermore, precision measurements were conducted on 60 instances of a standard mass, which was a stainless steel mass piece shaped like a Mercedes badge. Referred to as the “standard mass piece,” this was subjected to the same relative humidity (RH%) and temperature ranges as the filter samples.

The reference filter was an unexposed new filter that was retrieved from the packaging and allowed to equilibrate at 30%, 45%, or 55% RH for a 24-h period before measurements were conducted.

The primary influence on measurement accuracy is expected to be exerted by water vapor upon the filter, as opposed to alterations within the balance comparator. To scrutinize this influence more closely, a series of replicable measurements (*N*=10) were undertaken for each filter type (Table 3). Measurements were taken for the standard mass piece both individually (Table 2) and in conjunction with a reference filter (Table 3), across varying relative humidity (RH%) conditions. The aim of measuring the standard mass piece was to explore repeatability. The aim of adding the reference filter was to illustrate how shifts in RH conditions impact the mass of the reference filter.

The repeatability of the standard mass measurements is presented in Table 2. This also shows the impact of different humidity conditions on the deviation of the mass for the standard mass piece.

The table includes variables such as the average reference mass and RH% during manual and robotic measurements, along with statistical values like the mean, minimum, maximum, and standard deviation. The total mass of the standard mass piece exhibited a range of 149.81 ± 149.82 mg under the whole range of tested humidity conditions. This implies that, under the hypothetical assumption of “constant” conditioning parameters close to those outlined in the EN12341:2014 standard, variations in filter mass occurred solely as a result of inherent filter mass variability and random errors (balance indication repeatability). This error fell within the range of ±1 μg, ±1 μg, and ±2 μg respectively for 30, 45, and 55% relative humidity during manual measurements and ±1 μg, ±1 μg, and ±1 μg for 30, 45, and 55% relative humidity during the automatic measurement, which closely aligned with the repeatability defined by the device manufacturer, typically 1–2 μg (Radwag.com), with a coefficient of variation from 0.04 to 0.13%. Slightly lower stability was observed under 55% RH compared to 30%.

Generally, the precision of measurements should change only as an effect of water vapor influencing the filter, not because of the changes inside the balance comparator. The electronic balance UMA 2.5Y.FC is equipped with an automatic calibration system that ensures precise measurement accuracy. The internal calibration standard is performed by the built-in standard mass. This testing assumes that the laboratory conditions affect the internal weight to the same degree as the tested material. To gain a better understanding of this effect, a series of repeatable measurements (*N*=10) were conducted for each filter type, which will be subsequently referred to as reference filters. These reference filters consist of unexposed glass, quartz, and PTFE filters conditioned for 24 h under 30%, 45%, or 55% relative humidity conditions. Each filter was placed individually alongside a standard mass piece on the balance.

Table 3 presents the total effects of humidity conditions under robotic and manual weighing for the reference filters and the standard mass piece placed together onto the balance. It was shown that, under conditions of 30–55% RH, the maximum difference in the standard and reference filters’ mass was in repeatable measurements and it was 4 μg (glass filter, 45% RH). Similar results regarding the reference filter mass were obtained no matter which method was used. Under manual and robotic measurement, the differences in mean mass for the standard mass piece plus reference filter were found at the fifth and sixth decimal places (therefore applied to μg mass changes). For example, the average difference between the manual and robotic measurements in the case of glass filters (G) was 23 μg, 3 μg, and 17 μg under 30%, 45%, and 55% RH, respectively; while for quartz it was 34 μg, 11 μg, and 51 μg. Similar differences were found for the PTFE O-ring measurements, equal to 5 μg, 20 μg, and 3 μg under the mentioned RH% conditions. It was clearly shown that the total difference in measurements including reference filters was higher compared to the standard mass piece, which suggests that the filter material has a much greater influence on the mass measurements compared to the effect only from the device. The highest comparability in the reference filters’ mass between the two methods was found for the PTFE O-ring filters, which can be explained by their hydrophobic characteristic.

## Results and discussion

The dataset consisted of approx. 720 significant measurements of filter masses. Some results from this dataset were excluded (as indicated when summing the number of observations in the histograms in Appendix 1). This was done because, for a few measurements, the mass difference between the first and second weighing did not meet the requirement of ≤60 μg recommended by the PN-EN 12341:2014 standard. Determining the impact of the variability of the equilibration conditions (humidity effect) on the mean mass and deviations of the loaded filter mass [mg] was started by analyzing the stability of the mass measurements during repeated weighing of the standard mass piece. This is a common practice performed in order to verify the accuracy and precision of the balance’s measurements. By weighing the standard mass piece 10 times (in each humidity condition), we assessed the balance’s precision and determined that the measurements were consistent. Based on the findings presented in Table 2, it can be observed that the differences in the mass of the standard mass piece under the typical equilibration conditions specified in the PN-EN 12341:2014 standard (45–55% RH and 19–21°C) were ±1–2 μg. Under the relative humidity of 30%, the mass of the standard mass piece was 149.81 mg with the difference in mass equal to ±1 μg, which is negligible. Consequently, it can be concluded that the fluctuation in the mass of the sampled PM filters that could be attributed to the error in the balance indication was in the range of ±1–2 μg, assuming that the stability of the balance under the equilibration conditions used in this study was very good. Of course, the standard mass piece is made of stainless steel, which is not easily affected by environmental factors such as humidity or temperature. This ensures that the mass remains constant, allowing for consistent testing of the balance’s repeatability.

The repeatability tests were performed not only for the standard mass piece but also for the so-called reference filters. Thanks to this simple experiment, we were able to check the influence of the filter media on the balance precision. The evaluation of the balance’s performance under the same conditions in the case of the reference filters showed that the manual balance and RWS provided the most consistent results for repeated measurements for the PTFE O-ring filters compared to the quartz or glass filters. This precision was found to be in the range of 3–20 μg, while for quartz, it was higher, accounting for 11–51 μg. By comparing these two methodologies (weighing the standard mass piece under diverse RH conditions and weighing the standard mass piece alongside the reference filter), it became apparent that the overall measurement disparity, encompassing the reference filter, exceeded that of the standard mass piece alone. This implies that the filter material exerts a more prominent influence on mass measurements than the effect solely attributed to the device. Particularly noteworthy is the fact that the greatest consistency in the reference filter mass between these two approaches was observed for the PTFE O-ring filters, probably owing to their hydrophobic properties. The assessment of the balance’s performance under identical conditions for the reference filters revealed that both the manual balance and RWS (robotic weighing system) yielded the most dependable outcomes for repeated measurements when dealing with the PTFE O-ring filters, in comparison to quartz or glass filters.

**Fig. 5:**
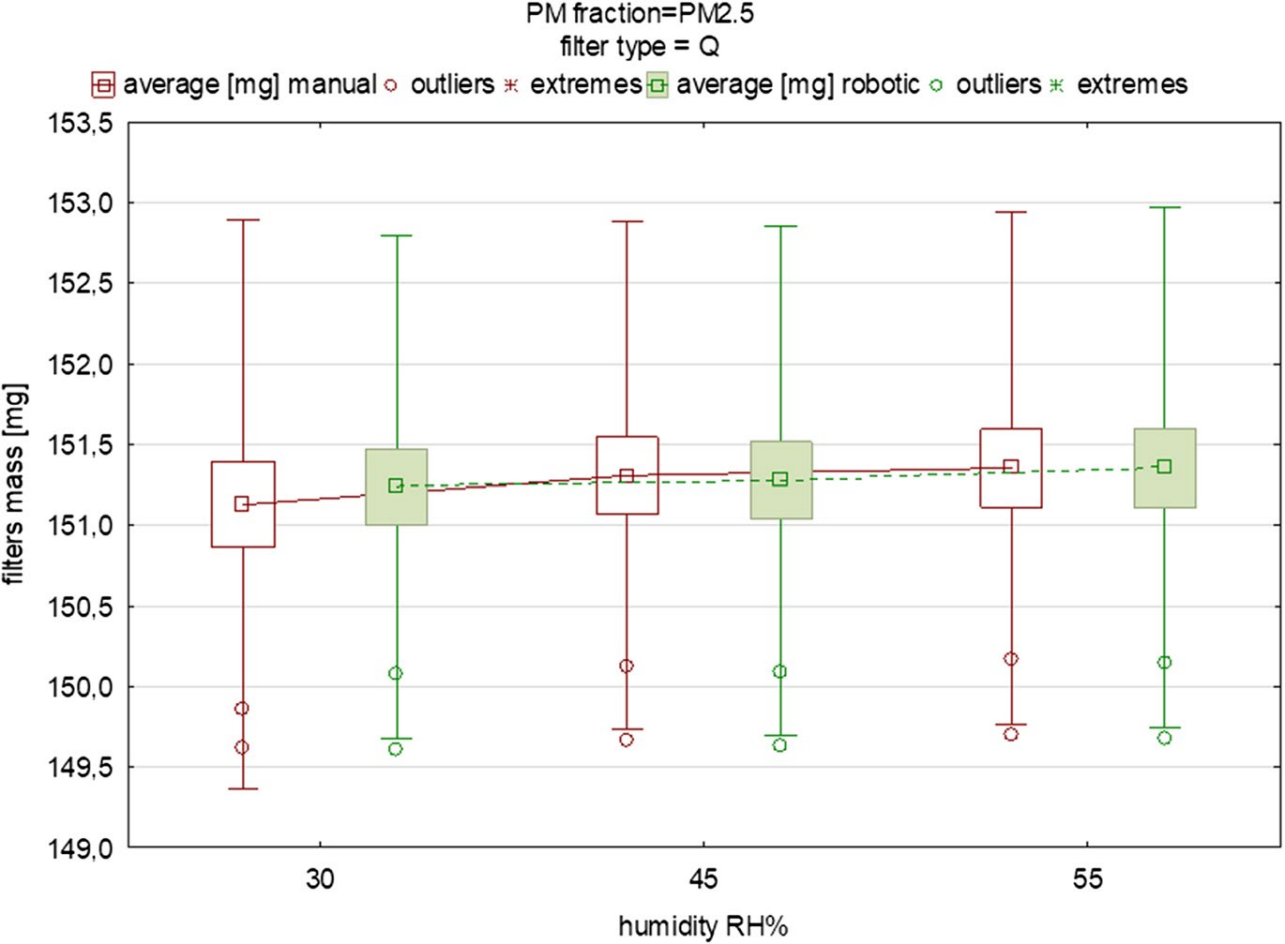
The influence of variable equilibration humidity on the mean mass and deviations of quartz filters loaded with PM_2.5_ (each box was drawn based on measurements of 15 filters from one batch)

**Fig. 6:**
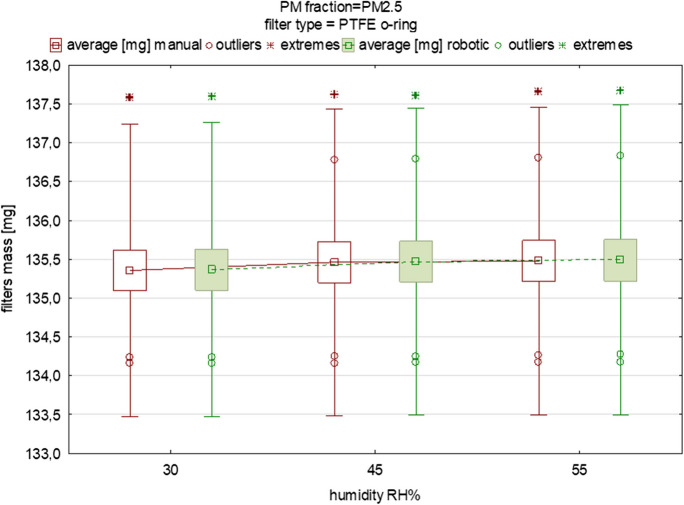
The influence of variable equilibration humidity on the mean mass and deviations of PTFE O-ring filters loaded with PM_2.5_ (each box was drawn based on measurements of 15 filters from one batch)

Figures 2, 3, 4, 5, 6 display the variability in the mass of filters loaded with PM_1_ and PM_2.5_ size fractions under humidity conditions in the range of 30–55% relative humidity (RH), including the mean values and the outliers range. These graphs also indicate the changes in the filters’ mass between the two different measurement methods. It is important to note that the predefined humidities presented in Figs. 2, 3, 4, 5, 6 should be treated only as “input” values. It is essential to remember that, while the robotic system maintains stable operating conditions within specific ranges, the actual temperature and moisture levels may deviate slightly from the set conditions. The actual humidity as well as the temperature inside the weighing room (in the case of the manual method) and inside the weighing chamber (in the case of the robotic one) is in fact slightly different compared to the input values. This discrepancy becomes evident when comparing the input temperature and humidity conditions in the experimental setups for both the manual and robotic weighing with the feedback from the sensors (the device-related uncertainty is given in the “Materials and methods” section). Humidity as well as temperature readings can be influenced by factors such as opening or closing doors when speaking about manual weighing, gusts of air caused by the flow of conditioned air into the gravimetric laboratory, or, for example (in the case of robotic measurement), the placement of sensors inside the weighing chamber and their device-specific uncertainty. Regarding the “sensors placement” factor, in order to quantify the influence of the arrangement of the sensors inside the chamber, we installed two additional temperature and humidity sensors in the corners of the chamber to be able to carry out research regarding the distribution of temperature and humidity conditions inside the RWS chamber in the near future. In this study, this effect was not studied.

Fig. 2 and Fig. 3 display the effect of humidity and the weighing method on discrepancies in the mass of the glass filters covered by PM_1_ and PM_2.5_ fractions. In the case of the PM_2.5_ fraction, the filters’ mass was higher, which is obvious when taking into account that the PM_2.5_ fraction includes fine PM_1_ particles. In almost all the tested subgroups, the mean of the filters’ mass was at the same level. Descriptive statistics regarding measurements of the location and dispersion of this variable are presented in Table 3. Although small differences in the average mass of the filters were observed, especially between 30 and 45% RH, the differences were not significant (*p*>0.05). The difference in the mean values of the filters’ mass under 30%, 45%, and 55% RH were 0.02, −0.05, and <0.00 mg, respectively. This means that the average difference in the mass of the loaded filters in those conditions between the robotic and manual weighing was not greater than 50 μg. A slightly lower difference was found for the PM_2.5_ fraction compared to PM_1_. This can be simply explained by the greater surface area of the PM_1_ particles, which probably take in atmospheric water vapor more effectively compared to the PM_2.5_ fraction, but also due to their chemical characteristics and mass size distribution, as determined in many previous studies (Klejnowski et al., 2012; Rogula-Kozlowska, 2015; Rogula-Kozlowska et al., 2017).

Across Tables 4, 5, 6, 7, 8, a notable increase in mass can be discerned for all three filter types within the 30 to 45% relative humidity (RH) range. However, in the transition from 45 to 55% RH, only the quartz filter exhibited a marked and statistically significant mass augmentation. Concerning the absolute mass and filter mass, the quartz filters exhibited the most pronounced hydrophilicity. The disparities in water content across the various temperature and humidity scenarios were also most pronounced in the case of quartz filters, as was presented in our previous study (Widziewicz-Rzonca & Tytla, 2020). This observation concurs also with the findings of Perrino et al. (2013), where an evaluation of quartz and Teflon filters for atmospheric water absorption during PM collection yielded similar outcomes. The inherent variability in filter masses, stemming from their production, leads to substantial variations in their weights. To determine whether these deviations are primarily a result of natural variability and to which extent humidity plays a role, we compared the mass under steady conditions (30% RH) with the filter mass under 45%RH and 55%RH humidity conditions (Figures A10–A12, Appendix). Our experience shows that, while natural variability is apparent among the 15 filters, the impact of humidity is still discernible and substantial.

**Table 8:** Results from Student’s *t*-test for dependent samples for testing significance of differences in average filter mass between manual and automatic measurement (PTFE O-ring filters, PM_2.5_)

Variable	Mean [mg]	Std. dev. [mg]	*N*	Mean diff. [mg]	Std. dev. diff. [mg]	*t*	df	*p*	Conf. int. −95%	Conf. int. +95%
PM fraction=PM_2.5_, humidity=30% PTFE O-ring filters										
Mass manual	135.36	0.94	13	−0.01	0.32	−0.10	12	0.9246	−0.20	0.18
Mass robotic	135.36	0.95								
PM fraction=PM_2.5_, humidity=45% PTFE O-ring filters										
Mass manual	135.46	0.99	14	−0.01	0.01	−2.56	13	**0.0239**	−0.02	−0.00
Mass robotic	135.47	0.99								
PM fraction=PM_2.5_, humidity=55% PTFE O-ring filters										
Mass manual	135.48	0.99	14	−0.01	0.00	−4.50	13	**0.0006**	−0.02	−0.01
Mass robotic	135.49	1.00								

bolded values are significant with *p*<0.05

Similar differences were found when testing the quartz fiber filters for PM_1_ and PM_2.5_ (Figs. 4, 5). The averaged difference in mean mass for the batch of PM_1_ filters was 0.02 mg and for PM_2.5_ it was 0.03 mg, suggesting a difference of approx. 20 and 30 μg between the manual and robotic indications (Tables 6, 7). For the PM_2.5_ fraction, a difference in mean filter mass between the two gravimetric methods was 0.11~111 μg (Table 7). This large difference was probably caused by the inaccurate placement of the filter in the holder of the RWS device rotor, possibly resulting in an incorrect measurement result (such a situation was observed for the third filter), but did not result in a significant difference (*p*>0.05). Significant differences were found under the 45% RH conditions when comparing automatic and manual weighing (*p*<0.05) (Tables 5, 6, 7, 8). This result should be studied further, however, especially at 50% RH such differences did not appear. The last comparison between manual and robotic weighing refers to the PTFE O-ring filters, which are most often used for PM sample collection in the USA air monitoring network. They are characterized by a high collection efficiency, chemical inertness, low background levels, and heat resistance, but also easy handling and compatibility. PTFE O-ring filters have gained acceptance in air quality monitoring practices and are recognized by US regulatory bodies. Their use is supported by standardized protocols and methods established by organizations such as the US Environmental Protection Agency (EPA). In Poland, routine use is made, instead, of quartz, and sometimes also glass filters.

In this study, only the PM_2.5_ fraction collected on PTFE filters (Table 8) is presented, while the PM_1_ fraction was collected on PTFE cellulose–supported filters. The reason for this choice was the behavior of PTFE-supported filters when exposed to particles. Unlike the PM_2.5_ filters, which remained flat, the PTFE-supported filters underwent a change in form, causing their corners to rise and adopt a more “U” shape instead of staying flat. Therefore, to ensure consistency and comparability in the study, the researchers decided to focus on the PM_2.5_ fraction collected on the PTFE filters, which maintained their flat shape throughout the sampling process. To address this issue, a simple solution was implemented. We folded the corners of the PTFE-supported filters to the opposite side while holding them with tweezers. This folding action helped bring the filters closer to their original flat shape, resulting in a larger contact surface when they were placed on the measuring element of the manual balance. By performing this procedure, the masses of the filters remained stable instead of fluctuating constantly. However, the challenge became more difficult to overcome during the robotic weighing. The main problem arose during the period when the filters were in the RWS (robotic weighing system) magazine. In the RWS, all 15 filters were positioned in a circular-shaped magazine, and they were measured one after another, without the option of adjusting the filter form immediately before the measurement, as in the manual measurements. Consequently, the challenge of maintaining a consistent filter form became more significant in the robotic weighing process. Some trials were done to adjust the filter shape while putting the filters into the magazine, but while the RWS is still scanning, the conditioned filters begin to lose their shape and when the measuring element arrives, the filters’ distorted form means that they cannot be stabilized on the measuring element, making the measurement results completely invalid. As in the case of quartz filters, statistically significant mass differences (*p*<0.05) were found for glass filters (PM_2.5_ fraction) under all the tested humidity conditions, for quartz filters under 45% RH, as well as for the PTFE filters (PM_2.5_ fraction) under 45% RH and 55% RH. These differences in the case of the quartz and glass filters were probably connected with a too-short conditioning time compared to the standard 48 h suggested (Widziewicz-Rzońca et al., 2022). Very often, especially when the filters are kept for a longer period of time in conditions significantly different from laboratory conditions, they need more time for equilibration. Another possible explanation for this phenomenon is water absorption, depending on the structure and material of the filter itself, but also on the chemical composition of the PM. The differences found mostly applied to the PM_2.5_ fraction, enriched in inorganic ions, since PM-bound water-soluble ions make up the greater part of the mass in PM_2.5_, especially in urban areas in the heating period (Rogula-Kozlowska et al., 2017). PM-bound water-soluble ions play a significant role in atmospheric chemical reactions, acting as precursors for new particles, especially sulfates (SO_4_^2−^), nitrates (NO_3_^−^), and ammonium (NH_4_^+^) (Błaszczak et al., 2019; Juda-Rezler et al., 2020), but also favor water sorption by PM. In the case of PM_2.5_ particles, secondary inorganic aerosol, responsible for the PM particles’ water affinity, may constitute as much as half of the PM_2.5_ concentrations at regional background stations (Guerreiro, 2013), and this can significantly affect the PM hygroscopicity, particularly during severe haze events with high RH% (Sun et al., 2020). Another explanation could be the numerical concentration of the tested PM particles. Previous studies performed in Zabrze indicate that approximately 99% of the particles in this location had aerodynamic diameters ≤1 μm, suggesting that the particles originate mostly from the combustion of fuels in domestic stoves or in car engines (Klejnowski et al., 2013). A numerical domination of fine and ultrafine particles, causing an increase in the sorption surface, will stimulate water condensation and mass addition. Another explanation could be the charging of the filters. Knowing that static charge decreases the accuracy of gravimetric analysis, one possible explanation, especially in the case of PTFE O-ring filters, is ineffective ionization by the antistatic stand (positioner) installed in the RB 2.4 YF weighing machine. To eliminate static charges on the filters, this device is equipped with a built-in antistatic frame, installed in the microbalance chamber. Our previous research leads us to suspect that nylon, polyamide, and PTFE filters require a longer charge reduction time. The information reported in the literature regarding the required neutralization duration to effectively diminish static charge to acceptable levels is inconsistent. In the study by Engelbrecht et al. (1980), the authors discovered that 47 mm Nuclepore filters remained inadequately neutralized even after a 30-s exposure, leading to potential bias of up to 150 μg attributed to static charge. Similarly, Allen et al. (1999) observed that, even with a 30-s exposure to 4 to 6 210Po sources, Teflon filters could not be weighed accurately due to measurement errors, with some cases experiencing errors exceeding 20 μg. In future research, we plan to lengthen the neutralization time and to investigate possible charging during weighing of the filters to look for this source of uncertainty. Research studies show that gravimetric methods are still preferable regarding air pollution monitoring with PM (Lagler et al., 2011). Therefore, nowadays, automation of the weighing process is being employed and more advanced instruments are being developed. These systems may include robotic arms or automated platforms that handle the filters, load them onto the balance, and record the mass measurements. The data can be stored electronically and integrated into data acquisition systems for further analysis. The use of automated balances for weighing PM filters offers several advantages over manual weighing, such as high precision and good repeatability, even at the level of microgram mass, which is most important when dealing with small amounts of particulate matter collected on filters, especially when analyzing samples collected in low contaminated areas. Manual weighing can be influenced by various factors, such as operator technique, environmental conditions, and human error. Automated balances help eliminate these inconsistencies by providing standardized and controlled weighing procedures. The automated process ensures that each filter is handled and weighed in the same manner, reducing measurement variability. Weighing a large number of PM filters manually can be time-consuming, especially if it is a routine task that needs to be performed regularly. Automated balances can weigh filters more quickly, increasing overall efficiency and allowing researchers to process a higher volume of samples in less time. According to the analysis conducted in 2016 by Presler-Jur et al., who examined a significant number of PM_2.5_ filters, the automated weighing method yielded results that were comparable to those obtained through the manual method, in terms of both accuracy and precision. This suggests that the automated method can provide reliable and consistent measurements, making it a viable alternative to the traditional manual weighing method for PM_2.5_ filters. In their study, the researchers conducted a comparison between manual gravimetric weighing and automated weighing using a robotic system. As part of their investigation, they also examined the impact of the human factor on the weighing results. The research findings led the group to conclude that the automated system successfully mitigated the variability in weighing results that can be caused by human factors. This improvement resulted in more consistent measurements. Additionally, the researchers demonstrated that the automated weighing process offered several other advantages over the manual method. Firstly, it led to faster processing times, so that the automated system could complete the weighing task more efficiently. Moreover, the automated process reduced the labor requirements by minimizing the involvement of researchers. This reduction in manual labor is beneficial as it alleviates both the physical and mental workload placed on researchers. By automating the weighing process, we are able to eliminate the need for repetitive manual tasks, such as handling filters and recording measurements. This further streamlines the workflow, allowing a focus on other important aspects of research work. Overall, the adoption of automated weighing systems brings enhanced efficiency, consistency, and reduced workload to the measurement process. Automated balances can be connected to data acquisition systems or laboratory information management systems (LIMS) for seamless integration and data transfer. By establishing this connection, the weighing data from the automated balances can be directly transmitted to the data acquisition systems or LIMS (laboratory information management system), facilitating efficient data management and analysis. In certain cases, such as when using PNS samplers (MCZ, Umwelttechnik) with the Comde-Derenda Model AWS-1 (automatic weighing system), an RFID (radio-frequency identification) system can also be employed. The RFID system allows for the identification and tracking of individual filters or sampling media, enabling automated weighing and recording of data associated with each specific sample. This technology further enhances the automation and traceability of the weighing process. By leveraging these connectivity options, researchers can streamline their workflows, improve data accuracy, and reduce the potential for manual errors. The integration of automated balances with data acquisition systems brings efficiency and reliability to the weighing process in various laboratory settings and allows for seamless recording and storage of measurement data. It also facilitates data analysis, sharing, and retrieval in a digital format. While comparing the change in filter mass for five filters to the average mass of each corresponding filter on day 1, L'Orange et al. (2021) found that the average absolute mass change from day 1, compared to any given day, was 0.8 μg ± 0.5 μg (*N* = 125). This value was significantly lower than the 15 μg requirement set by the US EPA. Throughout the 35-day period of repeated measurements, the average mass change for each filter remained within 4 μg of the mass recorded on day 1, which clearly suggested that the AIRLIFT robotic system achieves the measurement repeatability necessary for air quality monitoring requirements. The results of this study indicated that the automated weighing method provided comparable accuracy and precision to the manual method. The automated system demonstrated consistent performance across different conditions and sample masses. It also showed advantages in terms of reduced human errors, faster weighing times, and improved data management. The research conducted by L'Orange et al. in 2021 provides compelling evidence regarding the significant number of gravimetric measurements collected over a substantial time span, specifically from May 2018 to October 2020. The impressive figure of 80,000 measurements exemplifies the potential of automation to enhance operations within a gravimetric laboratory, bringing about notable improvements in efficiency and productivity. Our study demonstrates that automated weighing systems can offer reliable and efficient alternatives to manual weighing of PM filters. However, it is important to consider the specific equipment and protocols used in each study, as the performance of different automated systems may vary. Additionally, validation and calibration of the automated systems are crucial to ensure accurate and traceable results.

## Conclusions

In the present work, a new and simple methodology was developed to accurately determine the mass of different PM size fractions deposited onto filter membranes by using manual and robotic methods. Remarkably, the study has revealed that fluctuations in humidity levels within the tested ranges of 30%, 45%, and 55% had only a minor effect on the mass variations of the standard mass piece, amounting to approximately 1–2 μg. These results signify the robustness of the mass measurement process under varying humidity conditions. The negligible influence of humidity fluctuations prompts a pertinent consideration of the buoyancy correction procedure in relation to these findings.

The observed insensitivity of mass variations to humidity variations implies that, at the scale of precision investigated in this study, the buoyant force resulting from the displaced air caused by humidity changes did not significantly contribute to the measured mass differences. Consequently, the need for extensive buoyancy correction adjustments might be reduced or even deemed unnecessary within the humidity ranges studied. However, it is essential to exercise caution when extrapolating these conclusions to broader applications. Different objects and experimental setups might exhibit varied sensitivities to humidity-induced buoyant forces. As such, while the present study shows promising outcomes regarding the influence of humidity fluctuations, prudent consideration of buoyancy correction techniques remains a cornerstone of accurate mass measurement, especially when dealing with objects of varying volumes and densities. The investigation suggests that, within the humidity ranges tested, the role of buoyancy correction in mitigating humidity-induced mass variations may be relatively minor due to the minimal impact observed. When laboratory workers implemented high labor quality control measures for manually weighed filters, the precision between the analysts and RWS was very high, while the repeatability between the two measurement methods was compatible. Using stability tests for reference filters, it was clearly observed that standard mass drift is due to the filter media, not due to the instruments, and was greater for quartz filters than for glass or PTFE O-ring filters. Based on a simple comparison of the standard deviation under repeated measurements of the filters’ mass after exposure, it was not possible to state which measurement method is more accurate. The overall differences in the filters’ mass measured by the two methods under 30–55% RH was not greater than 50 μg. Both the MYA 5.4Y.F (Radwag) microbalance and the automatic weighing system UMA 2.5Y.FC were characterized by a very good reading accuracy equal to 1 μg and a maximum standard repeatability of 1.6 μg (for MYA 5.4Y.F) and 1–2 μg (for UMA 2.5Y.FC). While manual weighing may still be suitable for certain applications or when dealing with a small number of filters, automated balances offer increased accuracy, efficiency, and consistency, making them a preferred choice in many research and monitoring settings. The choice regarding the method used varied depending on the specific requirements, the application, and the level of precision needed.

### Supplementary information

ESM 1 (DOCX 604 kb)

## Data Availability

The datasets generated during the current study are available from the corresponding author on reasonable request.
