# Supplementary material for: Automatic weighing system vs. manual weighing precision comparison in PM-loaded filter measurements under different humidity conditions
Source: Environ Monit Assess. 2023 Oct 31;195(11):1393. doi: 10.1007/s10661-023-11939-7 (PMC10618374; doi:10.1007/s10661-023-11939-7)
Supplement: Supplementary file 1 — (DOCX 604 kb) [file 10661_2023_11939_MOESM1_ESM.docx]

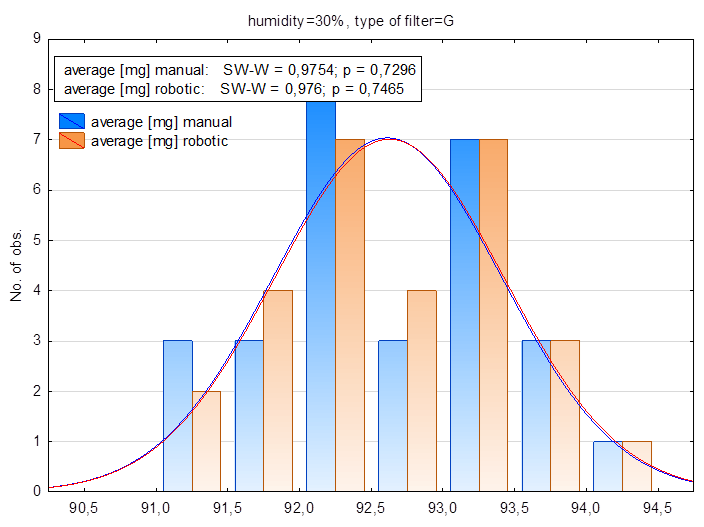


Fig A 1 Histogram with probability density curve and Shapiro-Wilk test (Glass filters, 30% RH)

Fig A 2 Histogram with probability density curve and Shapiro-Wilk test (Glass filters, 45% RH)

Fig A 3 Histogram with probability density curve and Shapiro-Wilk test (Glass filters, 55% RH)

Fig A 4 Histogram with probability density curve and Shapiro-Wilk test (PTFE o-ring, 30% RH)

Fig A 5 Histogram with probability density curve and Shapiro-Wilk test (PTFE o-ring filters, 45% RH)

Fig A 6 Histogram with probability density curve and Shapiro-Wilk test (PTFE o-ring filters, 55% RH)

Fig A 7 Histogram with probability density curve and Shapiro-Wilk test (Quartz filters, 30% RH)

Fig A 8 Histogram with probability density curve and Shapiro-Wilk test (Quartz filters, 45% RH)

Fig A 9 Histogram with probability density curve and Shapiro-Wilk test (Quartz filters, 55% RH)
